# Orthographic and Phonological Processing in Chinese Character Copying – A Preliminary Report

**DOI:** 10.3389/fpsyg.2020.02122

**Published:** 2020-09-15

**Authors:** Dustin Kai-Yan Lau

**Affiliations:** Department of Chinese and Bilingual Studies, The Hong Kong Polytechnic University, Kowloon, Hong Kong

**Keywords:** handwriting, writing, orthography, phonology, lexical processing, Chinese

## Abstract

In the current study, the effects of orthographic and phonological processing in Chinese character copying were investigated using a data set extracted from a database containing handwriting data of 856 stimuli; the responses of which were collected from 100 participants. To investigate the effect of character frequency, radical frequency, and phonetic regularity, 151 phonetic compounds were selected from the database because (1) their corresponding phonetic radicals were all free-standing characters, (2) their corresponding phonetic radicals were located at either the right or the bottom positions in the characters, and (3) no more than 10% of the participants made errors when copying these target characters. The results of the linear mixed effect models revealed that after controlling for inter-stroke distance (ISD) and stroke number, the inter-stroke intervals (ISIs) at the radical and logographeme boundaries were significantly longer, indicating significant orthographic processing in the immediate copying task that radicals and logographemes were used as writing units. In addition, shorter ISIs at the logographeme boundary associated with higher radical frequency, and shorter ISIs at the radical boundary associated with higher character frequency and regular characters, were observed. These observations indicated significant orthographic and phonological effects in the immediate copying task. Finally, the significant phonetic regularity effect observed also supported the notion that phonology contributes to Chinese character writing and that the effects of central processing, including character frequency and phonetic regularity, cascade over peripheral processing during Chinese character copying.

## Introduction

Numerous psycholinguistic studies have been conducted to investigate how people retrieve abstract orthographic codes from their mental lexicon and how they are converted into motoric execution in the process of handwriting. The architecture of the writing process can be divided into central and peripheral processing (Ellis and Young, 1996; Bonin et al., 2015). The central processes include orthographic long-term memory, conversion from phonology to orthography, and orthographic short-term memory. Orthographic codes retrieved from the central processing are externalized through peripheral processing, which includes allograph selection, graphic motor pattern selection, and graphic motor pattern execution. Previous psycholinguistic studies have also indicated that the extent to which different processes are involved in the lexical processing may vary according to the demands of different tasks (e.g., Inhoff et al., 1996; Bonin et al., 2015). For example, in an immediate copying task that the target stimuli is always available while the participants are writing the target words, the extent to which the writing process is affected by central processes such as orthographic and phonological factors may be minimal comparing with a writing-to-dictation task in which central processes should be heavily involved. In this study, the effects of orthographic and phonological factors on Chinese character copying were investigated.

Chinese is usually described as morphosyllabic in which each basic orthographic unit (i.e., character) corresponds to one syllable and one morpheme most of the time (Hoosain, 1992). For example, the character 言 corresponds to the morpheme <speech>, and the syllable (*jin4*; since this study was conducted in Hong Kong, where traditional Chinese characters and Cantonese are used, in this paper, phonetic transcriptions are represented in *jyutping*, a romanization system developed by the Linguistic Society of Hong Kong). The orthographic forms of Chinese characters are compilations of strokes organized in square constructions. One major type of Chinese characters, called phonetic compounds, contain semantic radicals that give clues meaning and phonetic radicals that give clues to phonology. For example, the character 評 (*ping4*) <criticize> can be decomposed into the semantic radical 言 <speech-related>, which indicates clues to its meaning, and the phonetic radical 平 (*ping4*) <flat>, which indicates clues to its phonology. Semantic and phonetic radicals were demonstrated in many studies to be involved in the decoding process of phonetic compounds (e.g., Feldman and Siok, 1999; Zhou and Marslen-Wilson, 2000; Law and Wong, 2005; Perfetti et al., 2005; Su et al., 2013; Lau et al., 2015). It has been widely reported that the recognition of regular phonetic compounds, those that share the identical syllables with their corresponding phonetic radicals, was associated with higher accuracy and faster reaction times (RTs) than responses to irregular phonetic compounds, those that do not share the identical syllables with their corresponding phonetic radicals (e.g., Su et al., 2013; Lau et al., 2015).

The roles of radicals in writing Chinese characters can possibly be 2-fold. First, the functions of individual radicals, i.e., the semantic and phonological information associated with the radicals, may affect the semantic and phonological processing in the writing process. Second, as frequently occurring orthographic units, they may affect the orthographic processing in the writing process.

Processing of semantic and phonological information associated with semantic and phonetic radicals is also evident in writing phonetic compound characters (e.g., Meng et al., 2000; Han et al., 2012; Lau and Ma, 2018; Lau and Yuen, 2019). For example, Law et al. (2005) tested a Chinese patient originally having normal writing abilities who suffered from dysgraphia, difficulties of writing, resulted from a stroke using writing-to-dictation and written naming tasks. They observed that in both tasks, the patient produced errors of additions, substitutions, and deletions of phonetic radicals and semantic radicals. These errors indicated that phonetic and semantic radicals are used as functional processing units in the Chinese writing process. Furthermore, Law et al. (2005) observed that instead of random substitutions, the semantic radical substitution errors produced by the patient were semantically related to the targets in the writing-to-dictation task. They suggested that the semantic radicals in one’s mental representations are linked to their corresponding semantic features. Hence, direct access to the semantic radicals in the orthographic lexicon from the semantic system is possible during Chinese character writing. In another study using a writing-to-dictation task with stimuli varied in terms of syllable-to-radical mapping consistency, Lau and Ma (2018) assessed a stroke patient with dysgraphia associated with semantic deficits. They reported that the patient demonstrated more preserved phonetic radicals when the syllable-to-radical mapping was consistent in the writing-to-dictation task. Similarly, Lau and Ma (2018) concluded that the mental representations of phonetic radicals are involved in the character writing process and they can be accessed directly *via* their corresponding syllables.

The processing of phonological information associated with phonetic radicals in writing is also in line with the trend of research that concerns whether phonology is involved in the process of orthographic code retrieval (e.g., Bonin et al., 2001; Zhang and Damian, 2010). Given the opaque relationship between phonology and orthography in Chinese, whether phonology plays a role in writing Chinese is of particular interest. The results have helped to verify whether, in the process of orthographic retrieval, contributions from phonology are language universal or language-specific. In fact, a growing body of recent research has reported the contributions of phonology in Chinese writing using writing tasks with primes (e.g., Qu et al., 2011). For example, in a written picture naming task with masked priming, Qu et al. (2011) manipulated the orthographic and phonological similarity between the primes and the target pictures. They reported a significant priming effect for primes that were orthographically and phonologically similar to the target pictures and primes that were phonologically similar but orthographically dissimilar to the target pictures, relative to primes that were neither orthographically nor phonologically similar to the target pictures. They concluded that phonology is involved in the process of writing. Similar results were reported in Qu et al. (2016), who also added that the priming effect disappeared when the written naming task was replaced by a manual semantic judgment task. Hence, they concluded that the priming effect originated from the orthographic output level.

In another study by Damian and Qu (2013), a written version of a Stroop task was used to investigate the involvement of phonology in written responses. They reported that slower RTs were associated with characters that shared the same syllables with a distractor color. That observation supported the finding that Chinese character writing is constrained by phonology.

As for orthographic processing in Chinese character writing, one of the most frequently asked questions concerns the grain size of the orthographic units involved in the processing (e.g., Law and Leung, 2000; Chen and Cherng, 2013; Lau, 2019b). In addition to semantic and phonetic radicals, there is another type of sub-lexical orthographic units frequently occurring in Chinese characters reported in the literature. Law and Leung (2000) documented a Chinese patient with dysgraphia who produced writing errors that involved substitutions of logographemes (i.e., stroke patterns in radicals that are spatially separated, such as “宀,”“八,” and “氵,” “土” and “土” in the radicals “穴” and “洼”, respectively). Han et al. (2007) also reported another patient suffered from stroke who produced similar errors of logographeme deletions, substitutions, and transpositions. These studies, therefore, provided evidence that besides radicals and strokes, logographemes are also functional processing units in writing Chinese characters. Since all the dysgraphic patients produced writing errors in all units of different grain sizes (i.e., strokes, logographemes, and radicals), Law et al. (2005) further suggested that representations of orthographic units with different grain sizes are all organized at the same level in the mental lexicon and are all involved in the writing process.

Distinguishing between logographemes and radicals, however, is not always straightforward. One reason is that some logographemes share the same orthographic forms with radicals. For example, the logographeme 木 in the character 堡 (bou2) <castle> shares the same orthographic form with the phonetic radical 木 (muk6) <wood> in the character 沐 (muk6) <bathe>. In the latter case, the radical 木 gives clues to the phonological form of the target character 沐, whereas in the former case, the logographeme 木 contributes to neither the sound nor the meaning of the target character 堡.

In this study, logographemes and radicals were defined according to whether they carry functions of semantic and phonological in the character contexts or not. Using the examples in the above, 保 (bou2) <protect> and 土 (tou2) <earth> are regarded as the radicals of the character 堡 because the former gives clues to phonology while the latter gives clues to meaning. The constituents of the radical 保 (i.e., 亻, 口, and 木) are regarded as logographemes.

With recent advancements in the technology of digital graphic tablets, an increasing number of studies have been conducted using handwriting measures to investigate the writing process (e.g., Kandel et al., 2012; Zhang and Feng, 2017; Lau, 2019a). Although the handwriting process, which includes the selection of allographs, retrieval of motor patterns, and execution of retrieved motor patterns, is usually considered peripheral processing in writing (Ellis and Young, 1996), recent studies have demonstrated that the effects of central processing, such as lexicality (Zhang and Feng, 2017), morphological complexity (Kandel et al., 2012), and script-to-sound regularity (Delattre et al., 2006), are reflected in handwriting measures. These observations support the notion that central processing cascades over peripheral processing during handwriting (e.g., Delattre et al., 2006; Qu et al., 2011).

For example, using a copying task involving suffixed and pseudo-suffixed words, Kandel et al. (2008) measured the inter-letter time intervals in the handwriting production of their participants. They observed that there were significantly longer inter-letter time intervals for suffixed than for pseudo-suffixed words. They suggested that the longer inter-letter time intervals were attributed to the extra processing time for the anticipation of the production of suffixes. The results they reported, being consistent with previous reports that supported the decomposed processing of morphologically complex words, provided support to the notion of central processing cascading over peripheral processing during handwriting (Kandel et al., 2008).

Applying similar measurements, Lau (2020) collected the inter-stroke intervals (ISIs), measured as the time difference between the end point of a stroke and the start point of the subsequent stroke, in the handwriting production by participants using a Chinese copying task. It was reported that after controlling for the inter-stroke distance (ISD; i.e., the linear distance between the end point of a stroke and the start point of the subsequent stroke), the ISIs located at the boundary between the semantic and phonetic radicals were longer than the ISIs within radicals. Furthermore, it was found that radical boundary ISIs of high-frequency characters were shorter than radical boundary ISIs of low-frequency characters. These observations are consistent with previous studies that have reported the radical boundary effect in peripheral processing (e.g., Zhang and Feng, 2017). A similar radical boundary effect was also reported in another study that involved school-aged participants (Lau, 2019b). In addition, Lau (2019b) found that the ISIs within radicals were significantly predicted by radical frequencies – ISIs within high-frequency radicals were shorter than ISIs within low-frequency radicals. The difference was attributed to the easier retrieval of graphic motor patterns associated with high-frequency radicals that are unique in Chinese writing (Lau and Yuen, 2019).

In the current study, similar handwriting measures were used to investigate the orthographic and phonological processing in a simple Chinese character copying task. To investigate the orthographic processing in copying Chinese characters, the participants’ use of orthographic units of different grain sizes was examined. The participants’ use of orthographic units of different grain sizes in copying Chinese characters was observed in two measures. In the first measure, ISIs located between orthographic units were compared with ISIs located within orthographic units. If orthographic units of a particular grain size are processed in copying Chinese character, the ISIs located before the orthographic units should be longer than the ISIs within the orthographic units. Therefore, to investigate if radicals are processed in copying Chinese characters, the ISIs located at the radical boundaries were compared with ISIs within radicals. Similarly, to investigate if logographemes are processed in copying Chinese characters, the ISIs located at the logographeme boundaries were compared with ISIs within logographemes. It was expected that similar boundary effect as reported in Lau (2020) would be observed if the participants used radicals and logographemes as the writing units in copying Chinese characters. In the second measure, the effect of frequency of occurrence of orthographic units of different grain sizes on ISI was observed. If orthographic units of a particular grain size are accessed during character copying, the effect of the corresponding frequency of occurrence should be significant in this experiment. Therefore, it was also expected that the between-radicals ISIs would vary as a function of character frequency as reported in Lau (2020). Similarly, it was also expected that the between-logographemes ISIs would vary as a function of radical frequency.

Finally, to investigate the phonological processing in Chinese character copying, the effect of phonetic regularity was observed using regular and irregular characters as stimuli. If the syllables associated with the characters and that of the phonetic radicals were involved in the processing, the mismatched syllables in the irregular condition compared with the shared syllables in the regular condition was expected to result in a competition effect. Hence, longer ISIs were expected to be associated with irregular characters relative to regular characters. Particularly, it was expected that if the syllables associated with the characters and syllables associated with the phonetic radicals are involved in Chinese character copying, longer ISIs at the radical boundaries (Zhang and Feng, 2017) would be associated with irregular characters.

To summarize, the aim of the current study was to investigate the orthographic and phonological processing in Chinese character copying using handwriting measures. To investigate the orthographic processing in Chinese character copying, the effects of radical boundary and logographeme boundary on ISIs, the effects of character frequency on between-radical ISIs, and the effects of radical frequency on between-logographeme ISIs were observed. To investigate the phonological processing in Chinese character copying, the effect of phonetic regularity on between-radical ISIs was observed.

## Materials and Methods

The data set reported in this study was extracted from the Database of Radicals in Written Chinese with Reliable Logographeme Boundaries (Lau, 2019a). In the database, a total of 856 traditional Chinese characters were selected from the Hong Kong Corpus of Chinese Newspapers (HKCCN; Leung and Lau, 2010) as the stimuli. There are 6,866 different traditional Chinese characters in the HKCCN, which consists of 123,677 news articles published by the eight most popular newspaper publishers in Hong Kong. Among the 856 characters in the database, there were 431 phonetic compound characters and 425 non-phonetic compound characters. Among the phonetic compound characters, there were 67 regular characters, 216 irregular characters, and 148 phonetic compound characters with bound radicals. A total of 100 right-handed undergraduate students (gender-balanced, mean age = 22.4 years, *SD* = 1.8) with normal or corrected-to-normal vision were recruited. All of the participants were native Cantonese speakers born and who received mainstream education in Hong Kong. None of the participants reported a history of cognitive, learning, or motor problems. Each participant was instructed to copy 172 characters from the total 856 stimuli in the database arranged in random order, such that for each stimulus, the handwriting data of at least 20 participants were collected.

### Stimuli

In the current study, 151 target characters were selected because (1) their corresponding phonetic radicals were all free-standing characters such that the regularity of the target characters could be defined, (2) their corresponding phonetic radicals were located at either the right or the bottom positions in the characters, and (3) no more than 10% of the participants made errors when copying these target characters. The following lexical and sub-lexical variables of the selected characters were also derived from the HKCCN.

#### Character Frequency

There are approximately 7.6 million characters in the HKCCN. The character frequency value of each of the target items refers to the counts of appearance of the character per million.

#### Radical and Logographeme Boundaries

**Figure 1:**
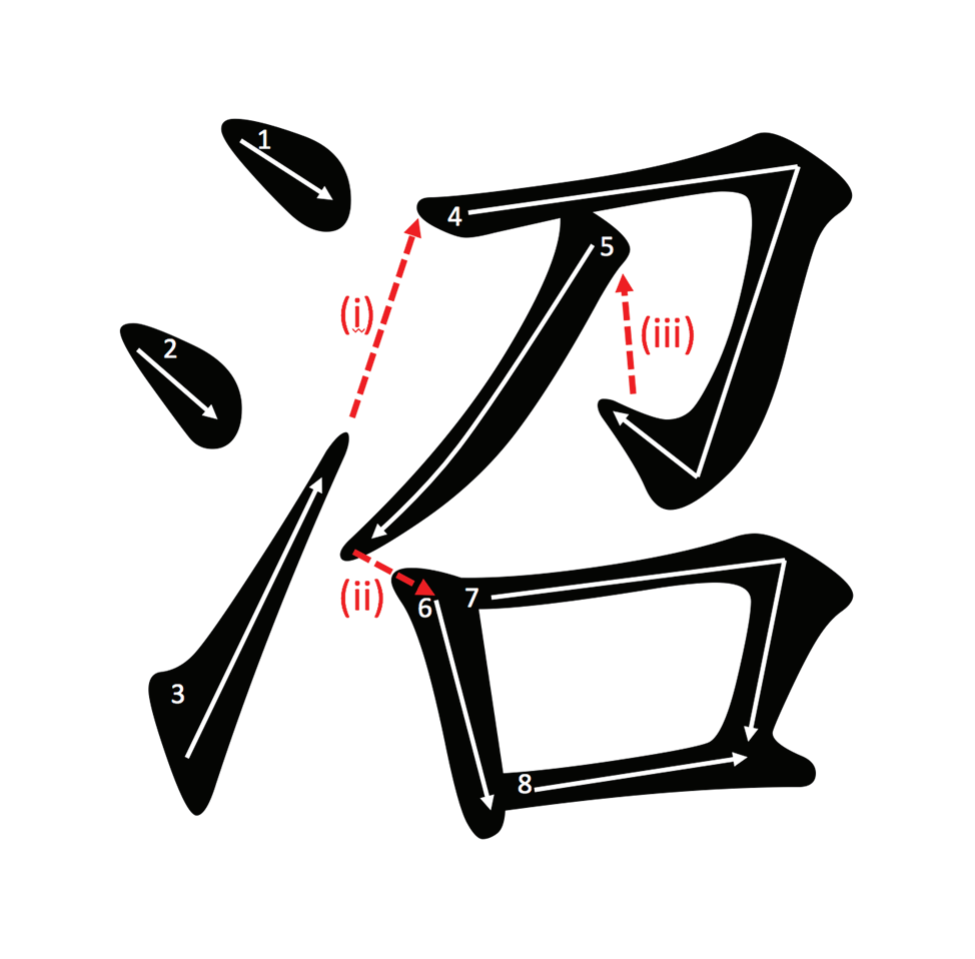
Example of different inter-stroke intervals (ISIs). **(i)** ISI at radical boundary, **(ii)** ISI at logographeme boundary, and **(iii)** ISI within logographeme. The Arabic digits denote the writing sequence.

Each ISI (and the corresponding ISD) obtained in the writing process was categorized into between-radical ISI, between-logographeme ISI, or within-logographeme ISI according to the position in which it occurred in the writing process. An example of the different ISIs is given in Figure 1. The constituent radicals and logographemes of the selected phonetic compound characters in the current study were defined according to the HKCCN. The semantic and phonetic radicals of the phonetic compound characters in the HKCCN were coded according to Xu (1963), while the constituent logographemes of the characters were coded according to Lui et al. (2010). According to Lui (2012), there was ambiguity in the process of logographeme identification, particularly when one identified logographeme was superimposed on another logographeme. Among all the selected characters in this current study, there were no superimposed logographemes to ensure that they had unambiguous radical and logographeme boundaries.

#### Radical Frequency

Radical frequency refers to the counts of different characters containing the corresponding radicals in the HKCCN. Radical frequencies of between-logographemes ISIs and within-logographeme ISIs refer to the frequencies of the radicals that they occurred in. Radical frequencies of between-radicals ISIs refer to the frequencies of the subsequent radicals. Using the example given in Figure 1, the radical frequency of the highlighted ISIs of (i), (ii), and (iii) refer to the radical frequency of the radical 召 (ziu6) <summon>.

#### Regularity

Characters that shared the identical syllables with their corresponding phonetic radicals were categorized as regular. Those that did not share the identical syllables with their corresponding phonetic radicals were categorized as irregular.

Table 1 summarizes the average character frequency, the first and second radical frequency, and the number of strokes in the regular and irregular groups of stimuli.

**Table 1:** Demographic information of the regular and irregular stimuli and *p* values of *t*-test comparisons.

	Regular	Irregular	*p* value
*N*	51	100	
Mean number of strokes (*SD*)	13.10 (3.63)	12.89 (4.64)	0.78
Mean character frequency^#^ (*SD*)	89.20 (141.91)	128.93 (172.46)	0.16
Mean first radical frequency (*SD*)	145.80 (116.79)	115.69 (103.88)	0.11
Mean second radical frequency (*SD*)	5.35 (10.71)	8.22 (13.01)	0.18
Mean number of logographemes (*SD*)	4.86 (1.34)	4.58 (1.68)	0.30
Mean age of acquisition (*SD*)	4.60 (0.87)	4.34 (0.92)	0.09

# Frequency values were counted in times/million.

#### Age of Acquisition

Age of acquisition (AoA) of each target character was obtained from the Canto-Lexicon Project (Lau et al., 2019). In the Canto-Lexicon Project, average AoA ratings obtained from 20 undergraduates (gender-balanced, with no prior linguistic training and literacy problem reported) of 4,376 most frequently found traditional Chinese characters were reported.

### Equipment

In the copying task, 7-in tablets with a resolution of 1,820 × 1,200 and a refresh rate of 60 Hz were used in this study. The tablets were quad-core with 2.20 GHz processing speed and they ran the latest Android 4.1.1 version. The tablets were installed with a homebrew Android application that controlled the display of the stimuli and recorded the written responses of the participants using the open-sourced MotionEvent package.

### Procedure

An immediate copying task was used. Each participant was instructed to use one tablet and one stylus pen in the copying task. Two pre-experimental training trials on using the stylus pen to write on the tablet were conducted to ensure that the participants knew how to manage the pen and tablet. In the randomly ordered experimental trials, a target character was displayed and the participants were required to directly copy the presented character on the tablet screen using the stylus pen. The target character remained on the screen during the entire writing process. The participants were instructed to write each stroke precisely by avoiding merging successive strokes. The elapsed time and coordinates each time the stylus pen touched or left the tablet screen were recorded accordingly. The duration of the whole experiment was about 15 min.

### Measures

The ISIs and the corresponding ISDs were calculated based on the coordinates, where the stylus pen left and retouched the tablet screen. Finally, the entire writing process and the final written output were also obtained.

### Data Analysis

Linear mixed effect models with maximal model structure (Barr et al., 2013) were computed using the lme4 package (version 1.1-18.1; Bates et al., 2015) in R (version 3.5.1; R Core Team, 2018) using the ISIs obtained. The ISD, AoA, stroke numbers, character frequency, radical frequency, logographeme frequency, and regularity of the corresponding ISIs were entered as fixed factors to investigate their significance in predicting the ISIs. By-subject and by-item random intercepts and random slopes were included for each fixed main effect based on recommendations by Barr et al. (2013). All frequency measures and stroke numbers were log-transformed to correct for skewness. Significance was determined using a cut-off point of *t* > 2. The results of the statistical models are summarized in Table 2.

**Table 2:** Results of the model examining the predictors of the ISIs.

Fixed effects	Estimate	*SE*	*t*		
(Intercept)	184.67	12.78	14.45		
Inter-stroke distance	0.53	0.01	55.56		
Age of acquisition	−0.43	1.20	−0.36		
Stroke number	14.85	2.77	5.37		
Radical_boundary	69.90	9.24	7.57		
CF	−12.87	2.42	−5.31		
Logographeme_boundary	29.97	1.71	17.53		
RF	−8.15	1.13	−7.22		
Regularity	−33.59	10.38	−3.24		
Radical_boundary: CF	11.36	2.43	4.68		
Logographeme_boundary: RF	8.13	1.16	7.03		
Radical_boundary: regularity	29.29	10.02	2.92		
**Random effects**	**Variance**	***SD***	**Correlation**		
Intercept|Subject	9757	98.78			
Radical_boundary|Subject	4776	69.11	−0.97		
Regularity|Subject	1861	43.14	−3.94	0.36	
Radical_boundary: regularity|Subject	1237	35.17	0.41	−0.47	−0.98
Intercept|Item	2793	29.32			
Radical_boundary|Item	1826	20.25	−0.87		
Regularity|Item	5049	71.05	−0.79	0.40	
Radical_boundary: regularity|Item	1486	38.55	0.63	−0.33	−0.86

SE, standard error; CF, character frequency; RF, radical frequency.

## Results

Data from the items with ISIs beyond three standard deviations from the mean (a total of 0.6%) were excluded from the analysis. The results showed that the ISI increased with ISD (0.53 ± 0.01). The longer the ISD, the longer were the ISI. Besides, ISI also increased with stroke number (14.85 ± 2.77), meaning that longer ISIs were associated with characters with more strokes. Effect of AoA was not significant in predicting ISI.

### Orthographic Processing

**Figure 2:**
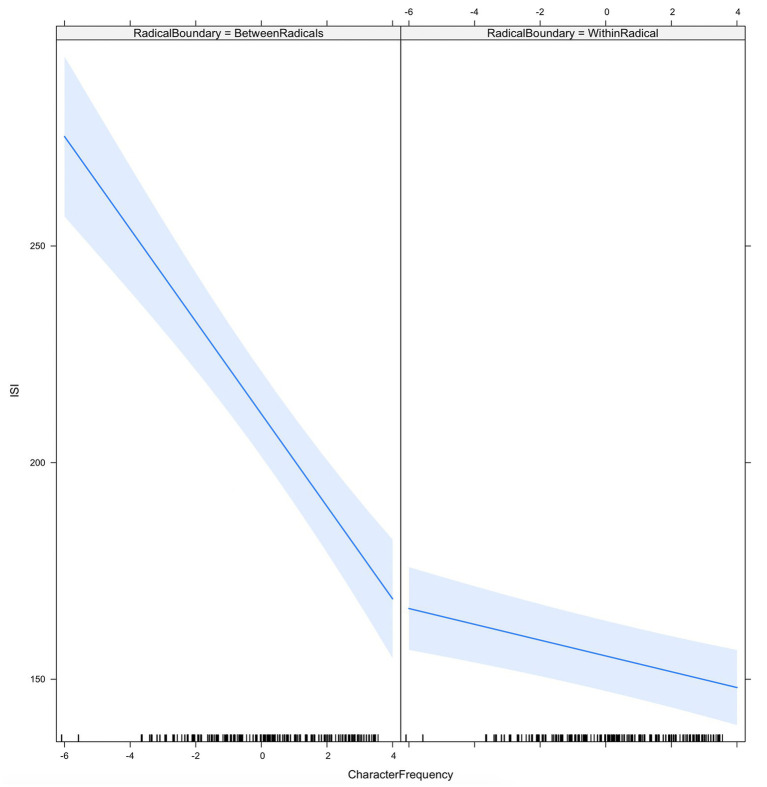
ISI as a function of radical boundary and character frequency.

Results showed that ISIs located at the radical boundaries were significantly longer than ISIs within radicals (69.90 ± 9.24, average count). Figure 2 shows the ISI as a function of radical boundary and character frequency. The results showed that the ISI decreased with character frequency (−12.87 ± 2.42, average count), particularly at the radical boundary (interaction of radical boundary/character frequency: 11.36 ± 2.43). As indicated in the figure, longer ISIs were observed at the radical boundaries among characters with lower frequencies.

**Figure 3:**
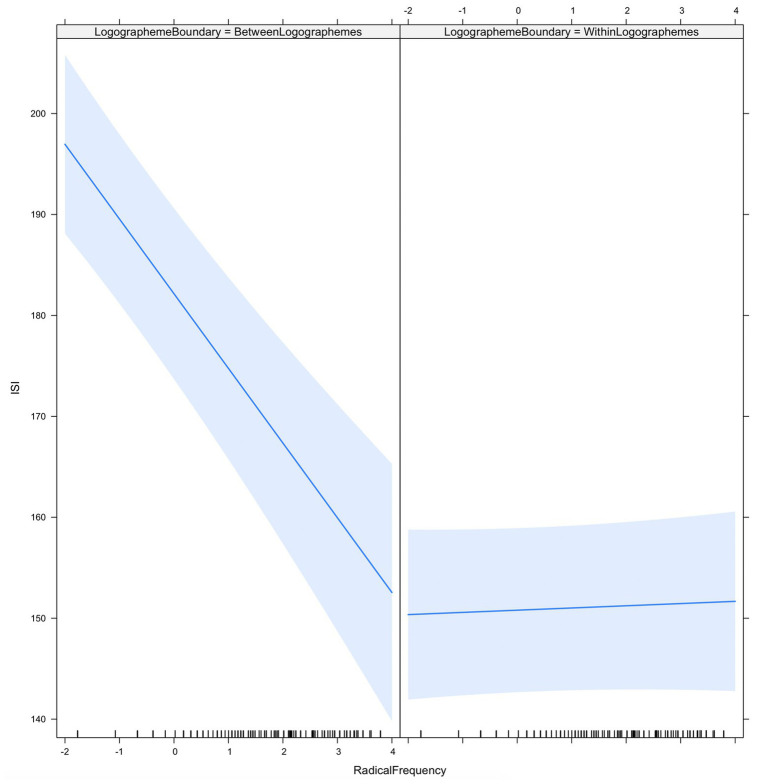
ISI as a function of logographeme boundary and radical frequency.

Results also showed that ISIs located at the logographeme boundaries were significantly longer than ISIs within logographemes (29.97 ± 1.71, average count). Figure 3 shows the ISI as a function of logographeme boundary and radical frequency. The results showed that the ISI decreased with radical frequency (−8.15 ± 1.13, average count), particularly at the logographeme boundary (interaction of logographeme boundary/radical frequency: 8.13 ± 1.16). As indicated in the figure, longer ISIs were observed at the logographeme boundaries among radicals with lower frequencies.

### Phonological Processing

**Figure 4:**
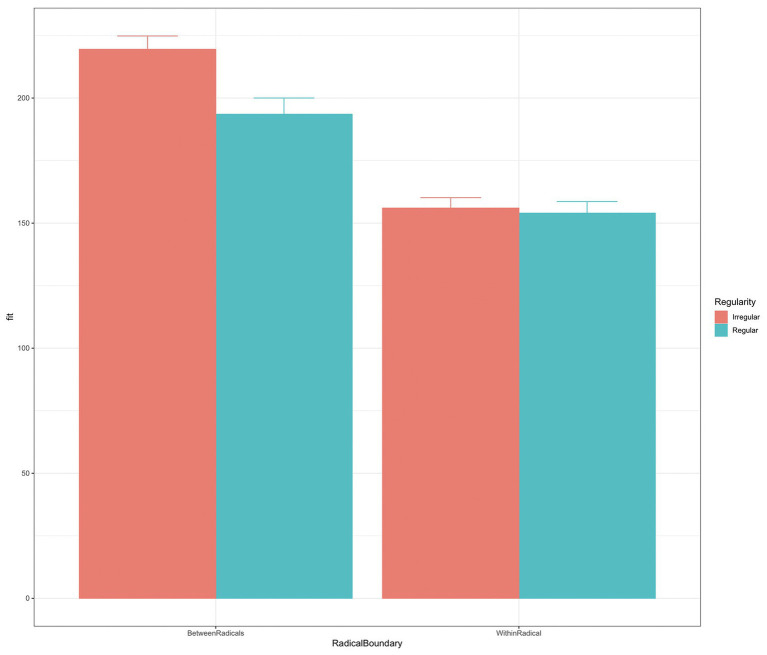
ISI as a function of radical boundary and regularity.

Results indicated a significant effect of regularity (−33.59 ± 10.38, average count). Figure 4 shows the ISI as a function of radical boundary and regularity. The results showed that the ISI decreased with regularity (−33.59 ± 10.38, average count) at the radical boundary (interaction of radical boundary/regularity: 29.29 ± 10.02). As indicated in the figure, shorter ISIs were observed at the radical boundaries among regular characters.

## Discussion

The current study obtained handwriting performance from participants using an immediate Chinese character copying task. The results showed that, consistent with previous handwriting studies (e.g., Lau, 2020), the ISDs were significantly associated with the ISIs in the participants’ handwriting performances. In addition, the results also showed that longer ISIs were associated with increased stroke numbers. This observation is consistent with previous handwriting studies that reported longer writing latencies associated with higher complexities of characters (Lau, 2020) and radicals (Zhang and Feng, 2017).

Unlike results reported in previous studies (e.g., Wang et al., 2020), the effect of AoA was observed to be insignificant in the current study. The insignificant AoA effect may be due to the strong correlations between character frequency and AoA, such that early acquired characters tend to be of higher frequencies, and the strong correlations between stroke number and AoA, such that early acquired characters tend to be less complex with fewer strokes. Given that character frequencies and stroke numbers are both strong predictors in the analysis, the effect of AoA may be diminished consequently.

The major aim of this study was to investigate the orthographic and phonological processing in Chinese character copying. The effects of radical boundary and logographeme boundary on ISIs, the effects of character frequency and phonetic regularity on between-radical ISIs, and the effects of radical frequency on between-logographeme ISIs were observed. The effects of these factors were discussed as follow.

### Logographeme Boundary

The significance of between-logographeme ISIs in the handwriting process supported the notion that logographemes are functional units in writing Chinese characters (Law and Leung, 2000; Han et al., 2007). Previous studies have attributed the effect to the longer processing time needed for the retrieval of the grapho-motor patterns of subsequent units and/or the planning for their execution in the writing process (Lau, 2020). This is also consistent with the suggestion that the graphic motor patterns of logographemes are represented in the brain for peripheral processing in writing Chinese characters (Lau and Yuen, 2019).

Moreover, it was observed that consistent with previous findings (Lau, 2020), longer between-logographeme ISIs were associated with radicals with lower frequencies. Therefore, the longer between-logographeme ISIs in low-frequency radicals suggest that the retrieval of the grapho-motor patterns of logographemes and/or the planning for their execution in the handwriting process was affected by the frequency of the radicals that the logographemes occurred in.

### Radical Boundary

The association of longer between-radical ISIs with lower-frequency characters is also consistent with previous findings (Lau, 2019b). Therefore, the longer between-radical ISIs in low-frequency characters suggests that the retrieval of radicals and/or the planning for their execution in the handwriting process was affected by the frequency of the characters that the radicals occurred in.

In addition to character frequency, the results also indicated that the between-radical ISIs were affected by the regularity of the characters. The results also indicated shorter between-radical ISIs of regular characters compared with that of irregular ones. This observation suggests that the syllables associated with the characters and that of their corresponding phonetic radicals were involved in the processing. In the irregular character condition, the syllables associated with the characters and the syllables associated with their corresponding phonetic radicals were different. The conflicts between the mismatched syllables probably resulted in the longer between-radical ISIs. On the other hand, no such conflicts existed between the matched syllables associated with the characters and their corresponding phonetic radicals in the regular condition. Hence, the between-radical ISIs were not affected in the regular condition.

### Orthographic Processing in Chinese Character Copying

The results indicated that despite the immediate copying task allowed a stroke-by-stroke approach, which requires minimal orthographic processing, in writing, the participants showed the tendency to break the target characters down into frequently occurring smaller constituents as processing units. The significantly longer between-radicals ISIs and between-logographeme ISIs supported this notion. This tendency to use radicals and logographemes as writing units was consistent with previous studies (e.g., Law and Leung, 2000; Lau, 2020). Besides, the current study further added that when there exists writing units with different grain sizes (i.e., characters, radicals, and logographemes), the choice of writing units depends on the orthographic frequency of the units. As indicated in the results, when the character frequency is low, longer between-radicals ISIs were observed. Similarly, when the radical frequency is low, longer between-logographemes ISIs were observed. These suggested that when the participants were required to copy large grain size chunks of low frequencies, they showed greater tendency to rely on smaller grain size chunks as writing units. On the other hand, when they were required to copy larger grain size chunks of high frequencies, such tendency to rely on smaller grain size chunks as writing units became smaller. These observations also supported previous suggestion by Law et al. (2005) that large and small grain size orthographic units are all organized in the orthographic representations at the same level in the metal lexicon and are all involved in the writing process, even in an immediate copying task which can be achieved with minimal lexical access.

### Contributions of Phonology in Copying Chinese Characters

Similarly, despite the task requirement of the immediate copying task can be fulfilled using a stroke-by-stroke approach without accessing to orthography and phonology, significant phonetic regularity effect was still observed, indicated that phonology is processed during character copying. It is important to highlight that if only the syllables associated with characters or syllables associated with the phonetic radicals were involved in the copying process, the phonetic regularity effect would not be significant. In order to explain the significant phonetic regularity effect observed, a mismatch between the syllables associated with the irregular characters and the syllables associated with the phonetic radicals have to be assumed. Therefore, the significant phonetic regularity effect observed indicated that both syllables associated with characters and syllables associated with phonetic radicals were involved in the character copying process.

The contribution of phonology in writing Chinese characters has been reported in previous studies using written picture naming tasks (Qu et al., 2011) and semantic judgment tasks (Qu et al., 2016). The current study added that the phonological effect was also observed in an immediate copying task. This significant phonological effect in Chinese character copying adds support to the suggestion of Bonin et al. (2015) that word copying relies on lexical pathways. Nevertheless, since phonetic regularity also affects character recognition, future work is needed to determine whether this phonetic regularity effect in copying Chinese characters was a result of the character recognition phase in the copying process or the post-recognition phase in the copying process.

### Were These Observations Driven by Central or Peripheral Processing in Chinese Character Writing?

The writing process can be divided into central and peripheral processing (Ellis and Young, 1996; Bonin et al., 2015). The orthographic long-term memory, conversion from phonology to orthography, and orthographic short-term memory are regarded as central processes. On the other hand, allograph selection, graphic motor pattern selection, and graphic motor pattern execution are regarded as peripheral processes.

The significance of the between-logographeme ISIs in the handwriting process observed in the current study supports the finding that the graphic motor patterns of logographemes are represented in the brain for peripheral processing in Chinese character writing. That the retrieval of the grapho-motor patterns of logographemes and/or the planning for their execution was affected by the frequency of the radicals in which the logographemes occurred further added complications to the significance of the between-logographeme ISIs in the handwriting process.

Two possible explanations are proposed. The first possibility is that both the graphic motor patterns of radicals and graphic motor patterns of logographemes are available in the mental representations and they can be flexibly used during the peripheral processing of Chinese characters writing. When radical frequencies are high, the graphic motor patterns of the radicals can be used directly for peripheral processing in character writing. On the other hand, when radical frequencies are low, they will be broken down into smaller grain size logographemes for peripheral processing in character writing. This explains the observed significant radical frequency effect at the logographeme boundary.

Alternatively, it is also possible that the significant radical frequency effect at the logographeme boundary reflected the cascading of central processing to peripheral processing in character writing (e.g., Delattre et al., 2006; Qu et al., 2011). According to Law et al. (2005), orthographic units with different grain sizes are represented at the same level in the orthographic mental lexicon and are all available and involved in the writing process. Therefore, it is possible that the shorter between-logographeme ISIs within high-frequency radicals were resulted from the higher efficiency of retrieval of higher-frequency radicals in the orthographic lexicon. As retrieval of radicals in the orthographic lexicon is part of the central processing, the observed shorter between-logographeme ISIs within high-frequency radicals is considered a reflection of cascading of central processing to peripheral processing in character writing.

An increasing number of studies have provided supportive evidence to the idea of a cascaded relationship between central processing and peripheral processing in writing (e.g., Roux et al., 2013). Previous studies conducted using handwriting measures have also reported significant effects of central processing (e.g., Kandel et al., 2008; Zhang and Feng, 2017). Zhang and Feng (2017) reported that people tend to slow down their handwriting execution at radical boundaries, which is an indicator of peripheral processing in writing. In the current study, it was observed that the radical boundary was also affected by the phonetic regularity of the characters. This observation suggests that the lengthened radical boundary in the handwriting process resulted from not only peripheral but also central processing. Nevertheless, it is suggested that more work is still needed to determine whether the association of longer between-logographeme ISIs with low-frequency radicals is a result of peripheral or central processing in writing.

### Limitations and Future Studies

It is important to point out that in the current study, the dataset was extracted from a large database for the experiment, including stimuli that did not belong to the regular and irregular categories. Unlike the typical factorial design in which only regular and irregular characters are included as stimuli, in the experiment, there were phonetic compounds and non-phonetic compounds in the stimuli set organized in random order. There were advantages and disadvantages to using the two different designs.

In a typical factorial design, the stimuli set is selected more carefully, such as matching the constituent phonetic radicals, the character frequencies, and stroke numbers in the two categories. This allows a relatively “purer” comparison between regular and irregular conditions. In the current design, although character frequency and stroke number between the two sets of stimuli were not statistically different, the group size and the set of phonetic radicals did not match. Hence, the comparison between regular and irregular conditions may not have been ideal. Future studies using stimuli matched perfectly in terms of constituent phonetic radicals, character frequencies, stroke numbers, and group size are recommended to further verify the findings of the current study.

Nevertheless, there was an advantage to using the current design. Because the selected items were embedded in a large set of different characters and the stimuli were presented in random order during the experiment, the unselected items actually served as fillers in the study. The regularity effect, therefore, was analyzed in a relatively more “natural” way in this study. Hence, one may expect that the regularity effect exists in natural Chinese character copying.

Finally, although there are certain limitations, e.g., the level of difficulties in the retrieval of orthographic representations is kept minimal, associated with the use of an immediate copying task to investigate the handwriting process, the potential of the application of the task in clinical settings, such as assessing individuals with developmental or acquired dysgraphia, should not be underrated. One common difficulty in studying dysgraphia concerns the high frequency of empty responses obtained in tasks involving high demands in cognitive and lexical processing, such as writing-to-dictation. As reported in the current study, using an immediate copying task, on the other hand, should allow the detections of certain effects associated with central and peripheral processing of writing among individuals with dysgraphia. Consequently, the possible difficulties underlying certain individuals’ writing problems can be hypothesized and tested accordingly.

## Conclusion

In the current study, orthographic and phonological processing in Chinese character copying were tested. Significant orthographic and phonological effects were observed in the immediate copying task, supporting the idea that word copying also relies on the lexical pathways of writing (Bonin et al., 2015). The significantly longer between-logographeme ISIs and between-radicals ISIs in the handwriting process supported previous findings that logographemes and radicals are used as processing units in writing Chinese characters (e.g., Law and Leung, 2000; Zhang and Feng, 2017; Lau and Yuen, 2019). The current study added that when there exist both large and small grain size processing units in writing, the choice of writing units depends on the frequency of occurrence of the units. In terms of phonological processing, the observation that the radical boundary was affected by phonetic regularity further added support to the notion that phonology contributes to writing in Chinese and the suggestion that central processing is cascaded to peripheral processing in writing Chinese characters.

## Data Availability Statement

All datasets generated for this study are included in the article/Supplementary Material.

## Ethics Statement

The studies involving human participants were reviewed and approved by the Human Subjects Ethics Sub-committee of the Hong Kong Polytechnic University. The patients/participants provided their written informed consent to participate in this study.

## Author Contributions

DK-YL is responsible for experiment design, data analysis, and preparation of the manuscript.

### Conflict of Interest

The author declares that the research was conducted in the absence of any commercial or financial relationships that could be construed as a potential conflict of interest.
